# Lipoprotein levels and statin treatment related to dementia and cognitive decline in individuals with type 2 diabetes: an observational analysis from the ADVANCE study

**DOI:** 10.1186/s12933-025-02894-3

**Published:** 2025-08-18

**Authors:** Peder af Geijerstam, John Chalmers, Miriam Pikkemaat, Ruth Peters, Michel Marre, Giuseppe Mancia, Mark Woodward, Katie Harris

**Affiliations:** 1 Primary Health Care Center Cityhälsan Centrum, and Department of Health, Medicine and Caring Sciences, Faculty of Medicine and Health Sciences, Linköping University, Linköping, Sweden; 2 The George Institute for Global Health, University of New South Wales, Sydney, Australia; 3 Center for Primary Health Care Research, Department of Clinical Sciences, Malmö, Lund University, Malmö, Sweden; 4 University Clinic Primary Care Skåne, Region Skåne, Malmö, Sweden; 5 Clinique Ambroise Paré, Neuilly-sur-Seine, France; 6 Institut Necker-Enfants Malades, INSERM, Université Paris Cité, Paris, France; 7 University of Milano-Bicocca, Milano, Italy; 8 The George Institute for Global Health, School of Public Health, Imperial College London, London, UK

**Keywords:** Dementia, Cognitive decline, Cholesterol, Diabetes, Diabetes complications, Lipids, Lipoproteins, Type 2 diabetes

## Abstract

**Introduction:**

Studies on the association between lipid levels and lipid-lowering treatment and the risk of dementia and/or cognitive decline (CD) have shown conflicting results and are few in individuals with type 2 diabetes (T2D). The aim was to evaluate the relationship of baseline LDL cholesterol levels and statin treatment with the development of dementia/CD in patients with T2D from the Action in Diabetes and Vascular Disease: Preterax and Diamicron Modified Release Controlled Evaluation trial.

**Methods:**

Dementia was diagnosed using the Diagnostic and Statistical Manual of Mental Disorders, 4th Edition (DSM-IV), and CD was defined as at least a 3-point decrement in the Mini Mental State Examination score. Exposures were baseline LDL cholesterol levels, statin treatment at baseline, and statin treatment initiation during the first 18 months of follow-up. Multinomial logistic regression was used to estimate the odds ratio (OR) and 95% CI for the composite of dementia/CD.

**Results:**

Of 11,140 participants, 1827 (16.4%) developed dementia/CD over the 5-year follow up. The OR (95% CI) of dementia/CD were 1.06 (1.00–1.14) per standard deviation higher in baseline LDL cholesterol and 0.90 (0.79–1.03) for participants with vs without statin treatment.

**Conclusion:**

We observed an association between LDL levels, but not statin treatment, and incident dementia/CD. Although causality cannot be determined by our study, the results are in line with multiple randomised controlled trials. However, to understand the long-term effects of lipid levels and statin treatment on dementia/CD, studies of longer follow-up are still needed.

**Supplementary Information:**

The online version contains supplementary material available at 10.1186/s12933-025-02894-3.

## Introduction

Elevated levels of blood lipids, including triglycerides and low-density lipoprotein (LDL), increase the risk of atherosclerosis and cardiovascular disease, while their reduction lowers the risk, especially in patients at high risk [1–3].

Up to a quarter of total body cholesterol is found in or near neurons [4]. Cholesterol is vital for brain development, and low HDL (but not LDL or triglycerides) has been associated with a smaller hippocampus, which is also seen in neurodegenerative disease [2]. However, the relationship between circulating lipid levels and brain development is disputed, as the blood–brain barrier is impermeable to cholesterol, and the cholesterol needed for brain function is produced locally [5]. There is no clear evidence that low LDL cholesterol levels as a result of lipid-lowering treatment would affect brain lipids [6]. In addition to vascular mechanisms, the ApoE ε4 allele may influence the relationship between lipid levels and incident Alzheimer's disease and all-cause dementia [7].

In meta-analyses, high midlife, but not late-life, total cholesterol has been positively associated with Alzheimer’s disease. HDL and LDL were not associated with dementia or cognitive decline (CD), but few studies reported relevant data [7, 8]. In cohort studies, overall results continue to be mixed with both HDL, LDL, and total cholesterol found to be associated with both subsequent increased and decreased risks of dementia and CD [5, 9–17]. Despite epidemiologic associations between lipid levels and dementia risk, clinical trials of statins have not demonstrated a protective effect against dementia or CD [4]. Trials on proprotein convertase subtilisin-kexin type 9 serine inhibitors and fibrates have shown mixed results [18–20]. On the other hand, in observational studies, associations between low LDL as a result of statin treatment and CD may be the result of confounding from pre-treatment hyperlipidaemia [1]. Observed associations between statin treatment and reduced incident dementia may also be the result of other residual confounders as well as reverse causation [21]. In randomized controlled trials on lowering of LDL cholesterol, the follow-up time observed may be too short and the participants too young, to capture any negative effects on cognition [1, 6].

In individuals with type 2 diabetes (T2D), large studies on hyperlipidaemia as a risk factor and the potential modifying effect of statin use on CD and dementia are few. T2D itself, as well as hyperlipidaemia, has been found to be associated with an increased risk of dementia, but findings are conflicting [4, 10, 22, 23].

## Aim

The primary aim of this study was to evaluate the relationship between baseline LDL cholesterol levels, measured before any treatment with statins, and the development of dementia and/or CD (dementia/CD) during follow-up in patients with T2D from the Action in Diabetes and Vascular Disease: Preterax and Diamicron Modified Release Controlled Evaluation (ADVANCE) trial. Secondary aims were to evaluate the relationship between statin treatment and the initiation of statin treatment, respectively, and the development of dementia/CD in the same population.

## Methods

ADVANCE was a randomized, controlled 2 × 2 factorial trial conducted in 215 centres in 20 countries in Asia, Australia, Europe, and North America between 2001 and 2008, which has previously been described in detail [24, 25]. In brief, 11,140 individuals with T2D aged 55 years or older and previous macro- or microvascular disease or at least one other risk factor for vascular disease were included. Individuals with a prior or current diagnosis of dementia were excluded from entry to the trial [26]. Participants were randomised using a factorial 2 × 2 design to a perindopril/indapamide combination blood pressure (BP) lowering treatment or matching placebo, and to gliclazide-based intensive glucose lowering therapy (target HbA1c ≤ 48 mmol/mol [6.5%]) or standard glucose control therapy based on guideline recommendations. The median follow-up duration was 5.0 years.

### Outcome

The primary outcome for the present study was a composite of dementia/CD. Cognitive function was evaluated using the Mini Mental State Examination (MMSE) at baseline and at two-yearly intervals during follow-up on 3 occasions, Supplementary Fig. 1. Original translated versions of the MMSE questionnaire were used; if a language was not available in the original version, a contextually appropriate translation of the MMSE was arranged. Cognitive decline was defined as at least a 3-point decrement in the MMSE score over any study interval. When an individual scored < 24 on the MMSE, or when the research physician or nurse coordinator suspected dementia, the individual was referred to a qualified specialist for a diagnosis based on the Diagnostic and Statistical Manual of Mental Disorders, 4th Edition (DSM-IV). The clinical assessment for dementia included an interview with both the patient and a close friend or relative, wherever possible. These clinical evaluation methods were standardized across all study centres. Both dementia and CD were prespecified secondary outcomes in ADVANCE.

### Exposures

Non-fasting venous blood samples were drawn at baseline. Total cholesterol, HDL-cholesterol, and triglycerides were analysed by local laboratories, and LDL-cholesterol was estimated using the Friedewald equation [27]. Information on medications were collected every 6 months, and statin treatment initiation during the first 18 months of follow-up was defined as present at 18 months and sustained until at least 24 months. If statin treatment was initiated but subsequently (at the next follow-up) terminated, the participant was regarded as never having initiated statin treatment.

### Statistical analysis

Baseline characteristics of participants were stratified by statin treatment as well as statin treatment initiation at baseline. Continuous data were summarised as mean (standard deviation) or median (inter-quartile interval) and categorical data as number (percentage).

The primary analysis was to evaluate the association between baseline lipid levels and incident dementia/CD. This analysis was restricted to participants without statin treatment at baseline, because in the entire cohort, low lipid levels would largely represent participants with statin treatment. The OR and 95% CI for each outcome during follow-up, together with the competing risk of death, was calculated per standard deviation higher in total cholesterol, HDL cholesterol, and LDL cholesterol levels at baseline, respectively. Two sets of model adjustment were fit: adjusted for age and sex (Model 1), and additionally adjusted for region, age at completion of highest level of education, randomised treatment group, baseline MMSE score, duration of T2D, waist circumference, smoking status (ever-smoker or never-smoker), alcohol intake (currently drinking at least once a week or not), systolic BP, estimated glomerular filtration rate (eGFR), urinary albumin-creatinine ratio, statin treatment at any time during the study period, and serum glycated haemoglobin level (Model 2). A sensitivity analysis was performed including all participants (even those with statin treatment at baseline). Unadjusted cubic spline curves were also used to graphically present the association of lipid levels with incident dementia/CD. The absolute risk of each outcome was illustrated using predicted probabilities with each respective lipid measure modelled as natural cubic splines with four degrees of freedom.

In the secondary analysis, to estimate the association of statin treatment vs no statin treatment at baseline on the composite of incident dementia/CD during follow-up, allowing for the competing risk of death, all participants were included. Adjustments were the same as above, except that in Model 2, serum LDL cholesterol was also adjusted for, and statin treatment during the study period was not adjusted for. The absolute risk of each outcome was estimated using multinomial logistic regression. Subgroup analyses were performed for both models of the secondary analysis and stratified by age (< 65 and ≥ 65 years), sex (male and female), and region of residence (Asia, Eastern Europe, and Established Market Economies) [28], respectively.

To estimate the association of statin treatment initiation, participants with statin treatment at baseline were excluded, and a comparison was made between those with vs those without statin treatment initiation during the first 18 months of follow-up. This analysis enabled adjustment for cholesterol levels at baseline, without any current lipid-lowering treatment affecting baseline values, and to only include participants with a known statin exposure period (as, otherwise, baseline statin exposure could be anything from a month to several years, because the US Food and Drug Administration approved the first statin in 1987 and the first generic statin in 1991) [29, 30]. Adjustments were the same as above.

To determine if there were any differences in the randomized treatment effect based on statins at baseline and statin initiation, the effects of active BP treatment vs placebo, and intensive vs standard glucose treatment on dementia/CD were assessed by two subgroups, (1) statin treatment at baseline, and (2) statin treatment initiation during the first 18 months of the study.

Three mutually exclusive endpoints were defined: (1) alive with neither dementia nor CD at the end of the study; (2) dementia/CD during the study, regardless of whether the participant died before the end of follow-up; (3) death preceding any dementia/CD during the study. Multinomial logistic regression was used to estimate the odds ratio (OR) and 95% CI, taking endpoint (1) as the reference. The individual component of incident dementia was also estimated using the same method. In a sensitivity analysis, participants without statin treatment at baseline, but with statin treatment initiation between month 0 and 18, were excluded.

Results were not materially different in models adjusted for multiple confounders as compared with age and sex adjusted analyses (Supplementary Figs. 3 and 4).

Statistical tests were two-tailed and *P* values of < 0.05 were considered statistically significant. R version 4.5.0 (R Core Team, Vienna, Austria) and RStudio version 2025.05.0 + 496 (Posit Software, Boston, MA, USA) were used for data analyses.

### Ethical considerations

The study was conducted in accordance with the Declaration of Helsinki. All participants gave written informed consent.

## Results

### Participant characteristics

Of the 11,140 ADVANCE participants, 3146 (28.2%) had statin treatment at baseline and, compared to those not on statin treatment at baseline, these were more often male (64 versus 55%) and from established market economies (69 versus 34%) (Table 1). However, those without statin treatment more often had insulin (1.6 vs 0.9%) and BP-lowering (13.5 vs 9.6%) treatment at baseline. Over the 5-year follow up, 1806 (16.2%) developed CD, 109 (1.0%) developed dementia, 1827 (16.4%) developed dementia/CD, and 1031 (9.3%) died, of which 929 (8.3%) without prior dementia/CD (Supplementary Fig. 2).

**Table 1:** Baseline characteristics of participants stratified by statin treatment at baseline and statin initiation at 18 months

	Statin treatment at baseline	No statin treatment at baseline		
		Total	Statin initiators at 18 months	Non-statin initiators at 18 months
N (%)	3146 (28.2)	7994 (71.8)	1027 (12.8)	6967 (87.2)
Age, years	65.8 (6.3)	65.8 (6.4)	65.7 (6.2)	65.8 (6.4)
Male, n (%)	2015 (64.0)	4392 (54.9)	567 (55.2)	3825 (54.9)
Region, n (%)				
Asia	458 (14.6)	3678 (46.0)	171 (16.7)	3507 (50.3)
Established Market Economies	2170 (69.0)	2692 (33.7)	651 (63.4)	2041 (29.3)
Eastern Europe	518 (16.5)	1624 (20.3)	205 (20.0)	1419 (20.4)
Ever-smoker, n (%)	1724 (54.8)	2950 (36.9)	490 (47.7)	2460 (35.3)
Alcohol intake once a week or more, n (%)	1271 (40.4)	2125 (26.6)	385 (37.5)	1740 (25.0)
Randomized BP-lowering treatment, n (%)	1538 (48.9)	4031 (50.4)	526 (51.2)	3505 (50.3)
Randomized intensive blood glucose control, n (%)	1554 (49.4)	4017 (50.3)	483 (47.0)	3534 (50.7)
Duration of diabetes, years	7.7 (6.4)	8.0 (6.3)	8.1 (6.5)	8.0 (6.3)
Co-morbidities, n (%)				
Atrial fibrillation	211 (6.7)	489 (6.1)	65 (6.3)	424 (6.1)
Microvascular complication	330 (10.5)	825 (10.3)	114 (11.1)	711 (10.2)
Macrovascular complication	1519 (48.3)	2071 (25.9)	262 (25.5)	1809 (26.0)
Medications, n (%)				
Blood pressure-lowering	301 (9.6)	1082 (13.5)	88 (8.6)	994 (14.3)
Anti-diabetic, any oral	2830 (90.0)	7259 (90.8)	921 (89.7)	6338 (91.0)
Anti-diabetic, insulin	28 (0.9)	131 (1.6)	10 (1.0)	121 (1.7)
Lipid-lowering other than statins	148 (4.7)	788 (9.9)	150 (14.6)	638 (9.2)
Body mass index, kg/m^2^	29.4 (5.1)	27.9 (5.2)	29.5 (5.3)	27.7 (5.1)
Waist circumference, cm	101.6 (12.5)	97.3 (13.1)	101.3 (13.1)	96.7 (13.0)
Laboratory results				
Plasma fasting glucose, mmol/L	8.2 (2.5)	8.6 (2.9)	8.4 (2.6)	8.6 (2.9)
Blood glycated haemoglobin, %	7.3 (1.3)	7.6 (1.6)	7.4 (1.5)	7.6 (1.7)
Blood glycated haemoglobin, mmol/mol	56.8 (14.3)	59.4 (17.9)	57.6 (16.0)	59.6 (18.2)
Estimated glomerular filtration rate, ml/min/1.73 m^2^	71.5 (20.6)	76.3 (22.1)	73.7 (21.8)	76.7 (22.1)
Plasma cholesterol, mmol/L	4.8 (1.1)	5.4 (1.2)	5.7 (1.2)	5.3 (1.2)
Plasma triglycerides, mmol/L	2.0 (1.3)	1.9 (1.3)	2.0 (1.3)	1.9 (1.3)
Plasma LDL cholesterol, mmol/L	2.7 (1.0)	3.3 (1.0)	3.6 (1.1)	3.2 (1.0)
Plasma HDL cholesterol, mmol/L	1.2 (0.3)	1.3 (0.4)	1.3 (0.3)	1.3 (0.4)
Increased albuminuria ^a^, n (%)	858 (27.3)	2403 (30.1)	279 (27.2)	2124 (30.5)
Systolic blood pressure, mmHg	144.3 (21.1)	145.3 (21.7)	146.3 (21.3)	145.2 (21.7)
Diastolic blood pressure, mmHg	79.9 (10.6)	80.9 (11.1)	81.0 (10.9)	80.9 (11.1)
Mini Mental State Examination, score	28.6 (1.8)	28.5 (1.9)	28.6 (1.7)	28.5 (2.0)

Data are presented as mean and standard deviation or median with interquartile interval (lower quartile, upper quartile), unless otherwise stated. Regions were Asia (China, India, Malaysia, and the Philippines), Established Market Economies (Australia, Canada, France, Germany, Ireland, Italy, the Netherlands, New Zealand, and the United Kingdom), and Eastern Europe (Czech Republic, Estonia, Hungary, Lithuania, Poland, Russia, and Slovakia)

Abbreviations: HDL, high-density lipoprotein; LDL, low-density lipoprotein

a. Urine albumin-creatinine ratio at or above 30 mg/g

### Primary analysis

**Fig. 1:**
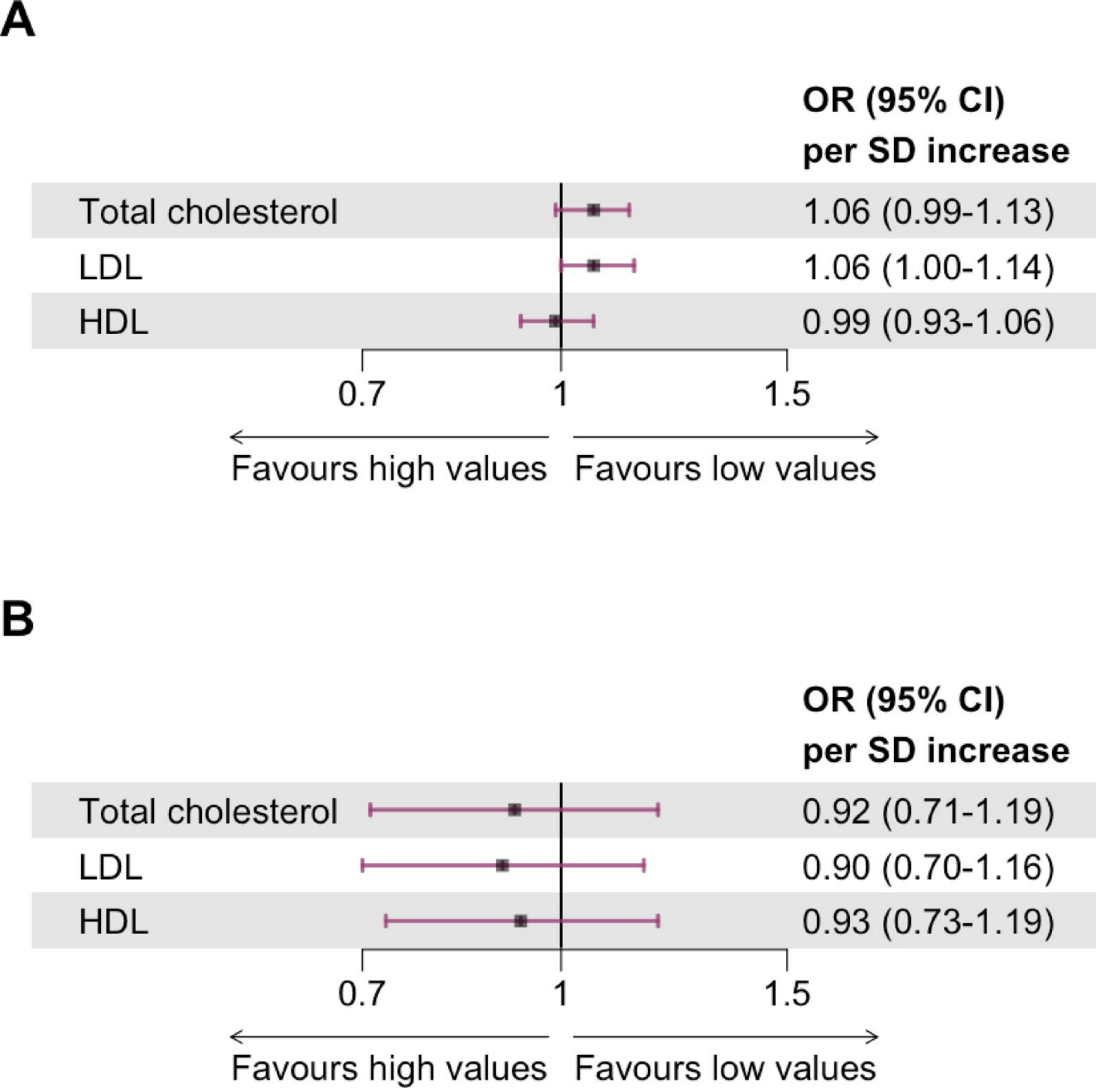
Forest plot of odds ratio and 95% CI of (**A**) dementia/cognitive decline and (**B**) dementia during follow-up per standard deviation increase in standardized baseline total cholesterol, low-density lipoprotein cholesterol, and high-density lipoprotein cholesterol, respectively, in participants without statin treatment at baseline after multiple adjustment. A multinomial logistic regression model was fitted to evaluate the association between baseline cholesterol levels and competing risks of death. Baseline cholesterol levels were standardized before analysis (mean-centred and divided by the standard deviation) to facilitate interpretation of results. Adjustments were for age, sex, region of residence, age at completion of highest level of education, randomised treatment group, baseline Mini Mental State Examination score, type 2 diabetes duration, waist circumference, smoking status, alcohol intake, systolic blood pressure, estimated glomerular filtration rate, urinary albumin-creatinine ratio, statin treatment at any time during the study, and serum glycated haemoglobin. Of 7994 participants without statin treatment at baseline included in analysis, 1363 (17.1%) had the outcome dementia or cognitive decline, and 83 (1.0%) had the outcome dementia. Abbreviations: HDL, high-density lipoprotein; LDL, low-density lipoprotein

**Fig. 2:**
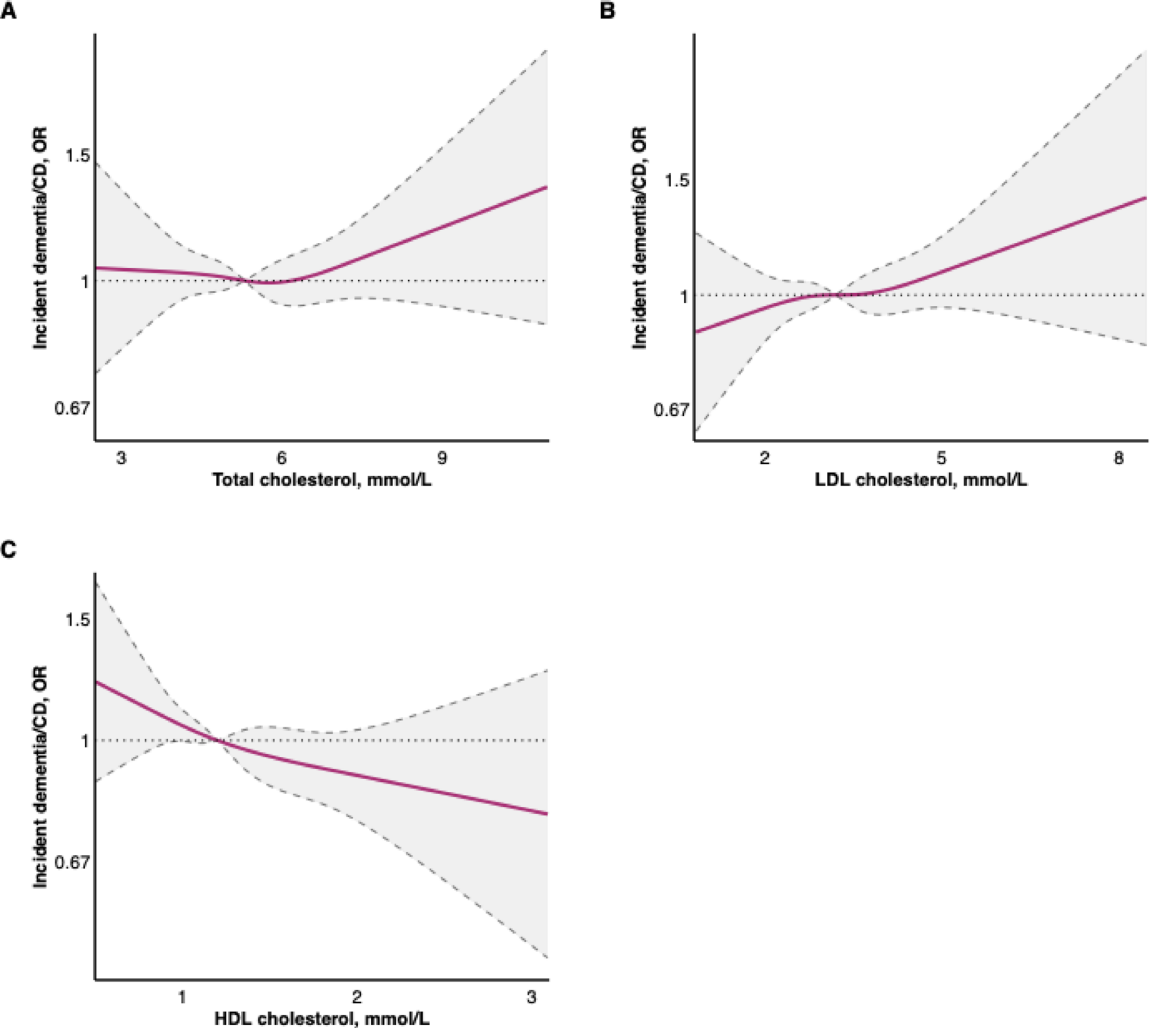
Cubic spline regression of baseline lipid levels and the odds ratio of incident dementia or cognitive decline. The model included a knot at the median value of each lipid: 5.3 mmol/L for total cholesterol, 3.2 mmol/L for LDL cholesterol, and 1.2 mmol/L for HDL cholesterol. Abbreviations: HDL, high-density lipoprotein; LDL, low-density lipoprotein

Per standard deviation higher, baseline LDL cholesterol, but not total cholesterol or HDL cholesterol, was associated with a higher odds of dementia/CD, OR (95% CI) 1.06 (1.00–1.14) compared with 1.06 (0.99–1.13) and 0.99 (0.93–1.06), respectively, in participants without statin treatment at baseline (Fig. 1A). This relationship was also evident as presented in cubic splines (Fig. 2). Of 7994 participants without statin treatment at baseline, only 83 (1.0%) developed dementia and results on baseline lipid levels were thus inconclusive (Fig. 1B). In a sensitivity analysis including all participants, these results did not change significantly (Supplementary Fig. 5 and 6).

### Secondary analyses

**Fig. 3:**
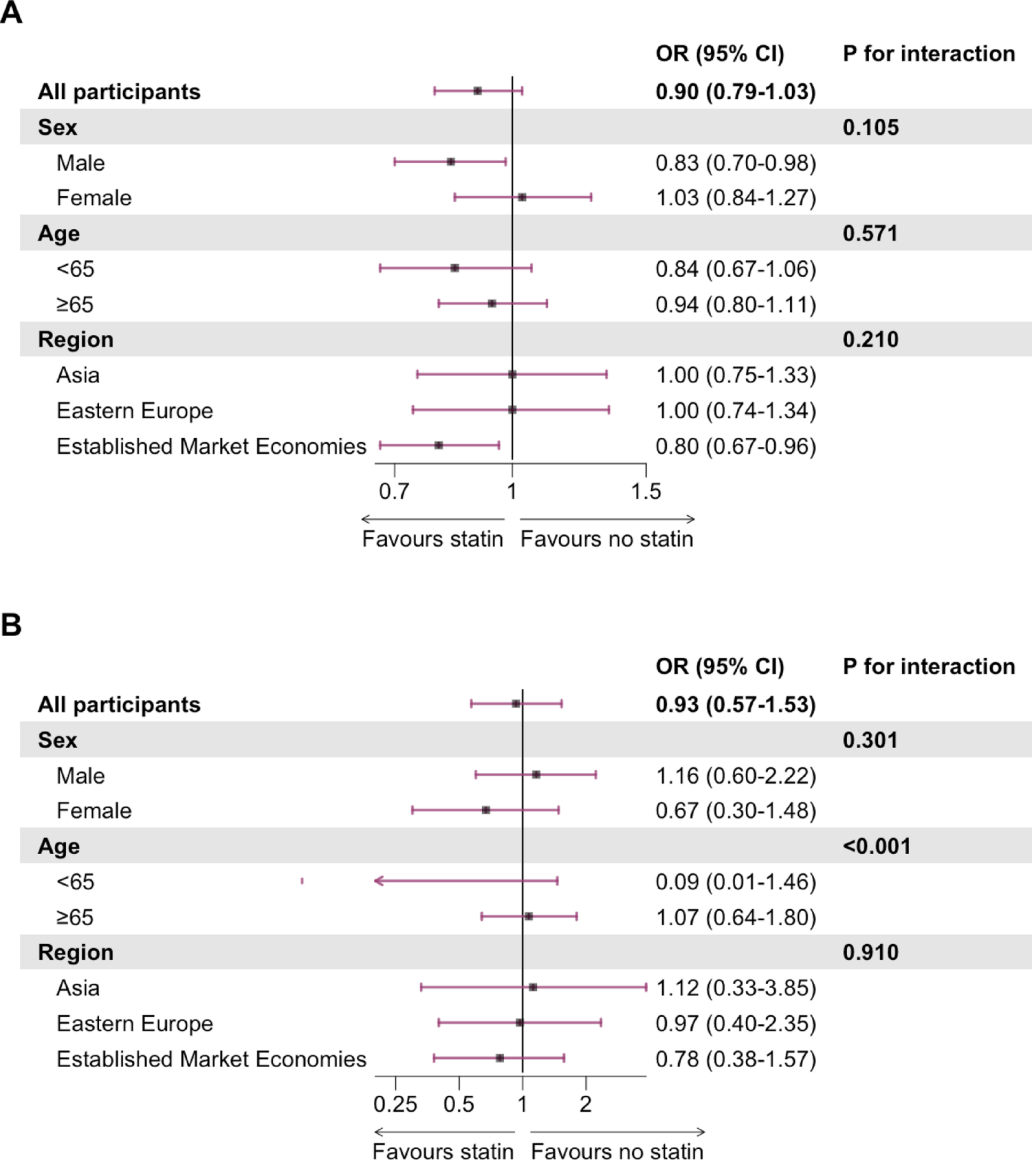
Forest plot of odds ratio and 95% CI of **A** dementia/cognitive decline and **B** dementia during follow-up for participants with statin treatment vs no statin treatment (reference) at baseline overall and by subgroups after multiple adjustment. Multinomial logistic regression with the competing risk of death adjusted for age, sex, region of residence, age at completion of highest level of education, randomised treatment group, baseline Mini Mental State Examination score, type 2 diabetes duration, waist circumference, smoking status, alcohol intake, systolic blood pressure, estimated glomerular filtration rate, urinary albumin-creatinine ratio, serum low-density lipoprotein cholesterol, and serum glycated haemoglobin. For analysis of male and female participants, respectively, adjustment for sex was omitted. Of 11,140 participants included in analysis, 1827 (16.4%) had the outcome dementia or cognitive decline, and 109 (1.0%) had the outcome dementia

The OR for dementia/CD and dementia, in participants with vs without statin treatment at baseline, were 0.90 (95% CI 0.79–1.03) and 0.93 (0.57–1.53), respectively, after multiple adjustments and allowing for the competing risk of death from other causes (Fig. 3). The risk of dementia/CD was 15.1 vs 17.0% in participants with vs without statin treatment at baseline (Supplementary Table 2).

After excluding participants with statin treatment initiation during the first 18 months of the study, none of the results changed significantly, but participants with vs without statin treatment at baseline showed a stronger tendency towards a lower risk of dementia/CD, OR 0.76 (95% CI 0.57–1.00), after multiple adjustment.

In participants with vs without statin treatment initiation during the first 18 months of the study period, the ORs for dementia/CD and dementia were 0.98 (95% CI 0.81–1.18) and 1.11 (95% CI 0.55–2.26), respectively, after multiple adjustment (Table 2).

**Table 2:** Odds ratio and 95% CI for cognitive decline and/or dementia during follow-up for participants with vs without statin treatment initiation (reference) during the first 18 months of the study period in participants without statin treatment at baseline

	Statin treatment initiation during the first 18 months		
	No, n = 6967	Yes, n = 1027	
		Adjusted for age and sex	Multiple adjusted
	OR	OR (95% CI)	OR (95% CI)
Dementia or cognitive decline, n = 1363	1 [Reference]	0.99 (0.83–1.18)	0.98 (0.81–1.18)
Dementia, n = 83	1 [Reference]	0.92 (0.47–1.79)	1.11 (0.55–2.26)

Multinomial logistic regression with the competing risk of death with multiple model adjusted for age, sex, region of residence, age at completion of highest level of education, randomised treatment group, baseline Mini Mental State Examination score, type 2 diabetes duration, waist circumference, smoking status, alcohol intake, systolic blood pressure, estimated glomerular filtration rate, urinary albumin-creatinine ratio, serum low-density lipoprotein cholesterol, and serum glycated haemoglobin

Of 7994 participants without statin treatment at baseline included in analysis, 1363 (17.1%) had the outcome dementia or cognitive decline, and 82 (1.0%) had the outcome dementia

There was no evidence of heterogeneity in the BP and glucose treatment effects by statins at baseline (*P* for interaction = 0.320 and 0.072, respectively) or statin treatment initiation during the first 18 months of the study (*P* for interaction = 0.677 and 0.392, respectively) (Supplementary Table 1).

## Discussion

In this study of 11,140 individuals with T2D, LDL levels in participants without statin treatment at baseline were associated with higher incident dementia/CD. Statin treatment was not associated with incident dementia/CD.

LDL cholesterol, but not total cholesterol or HDL cholesterol, was associated with a higher odds of dementia/CD. In a recent systematic review of 17 studies and more than 1 million participants, including studies using validated screening tools such as MMSE, each 1 mmol/L increase of LDL and total cholesterol was associated with an 8% and 1%, respectively, increase in incident dementia and mild cognitive impairment [31]. The association between LDL cholesterol and dementia was also seen in individuals using statins, both with and without diabetes, in a recent cohort study of more than 6 million individuals in South Korea followed for a median of more than 8 years. However, in statin non-users, LDL cholesterol levels in both the lowest and highest quartiles were associated with dementia [10]. Our findings indicate that in individuals with T2D and high CVD risk, as in the general population, LDL cholesterol is associated with dementia/CD. The association between cholesterol levels and dementia could relate to atherosclerosis and damage to the blood–brain barrier, as well as changes in the production, deposition, and clearance of amyloid beta and tau proteins [31, 32].

We did not find an association between HDL cholesterol levels and dementia/CD. Previous studies have reported an association between high HDL cholesterol and lower incident CD [2, 33]. Of these, one studied individuals with T2D and studies defined CD as MMSE < 27 points, which is similar although not directly comparable to ours [33]. The other study included individuals both with and without diabetes, and used MMSE as a continuous variable, thus increasing the power to detect more subtle changes than the 3 points we regarded as clinically significant [2]. Thus, differences in cohorts and methods may explain differences to our results.

Individuals with statin treatment at baseline were more often male (64 vs 55%) and from Established Market Economies (69 vs 34%). Such differences may reflect perceived cardiovascular risk on behalf of the clinician. However, several studies have also shown that women are less likely to be treated with statins than men, even though efficacy is comparable, because it is less often prescribed, and more often declined or discontinued [34, 35]. On the contrary, a study of individuals in low- and middle-income countries found higher statin use in women vs men, at least as primary prevention [36]. To further examine disparities in statin prescription patterns, including variations by sex and region, is a potential topic of future studies.

Individuals with vs without statin treatment at baseline showed a lower risk of dementia/CD, but after adjustments, this association was only seen in subgroup analyses of male but not female participants, and of participants from Established Market Economies but not from Asia or Eastern Europe. However, these associations observed in subgroup analyses were not confirmed in interaction analyses. Previous studies have raised concerns that statins could increase the risk of dementia, but randomized controlled trials of statin treatment have observed no deterioration or improvement in cognitive outcomes [1, 4]. However, trials were limited to at most 6 years, and studies with longer follow-up time have been called for [1]. In a recent meta-analysis of 46 observational studies, statin use was associated with lower incident dementia equally in both women and men [37]. In a UK biobank study, LDL cholesterol levels were associated with dementia in women but not in men, after adjustment for lipid-lowering drugs, which could implicate a benefit of statins for women rather than men [9]. Although the use of statins and the odds of dementia/CD was lower in female vs male participants in the ADVANCE cohort [26], we observed no pattern in the association between statin use and incident dementia/CD in female participants, indicating that it was not due to limited statistical power. Finally, we found no treatment effect modification of statins on dementia/CD, akin to the lack of treatment effect modification of statins on cardiovascular events previously shown [38].

Our study has several strengths. Data were from a large cohort of participants from several countries, which increases generalizability. Outcomes were independently adjudicated, and adjustments were made for several key confounders. Missing values for lipids was 1.1% or less. Finally, only 17 individuals were lost to follow-up over the 5-year study period. The study also has some limitations. Participants were not randomized to statin treatment and thus inference on causality cannot be made. Although plasma samples were non-fasting, fasting and non-fasting lipids, both with and without statin use, have been found to be comparable when predicting cardiovascular risk [39]. Participants had T2D and high CVD risk, and thus generalizability is limited to similar populations. Whilst we considered a clinical trial population, that might be perceived to lack generalizability, ADVANCE participants have been shown to be broadly generalisable to patients with T2D in community practice [40]. Immortal time bias was minimized by restricting exposure definition to the first 24 months, but bias may remain if early cognitive decline influenced statin initiation. Statin initiation was assessed at intervals, and MMSE was not measured at 18 months, possibly missing early cognitive decline and biasing results toward the null. The effects of LDL cholesterol levels and statin treatment may differ for different types of dementia e.g., vascular dementia and Alzheimer’s disease [41], but we did not have access to more granular data or genetic information to support further categorization or a more sophisticated understanding of dementia. We do not have data on fibrates (drugs that may increase HDL cholesterol), however other lipid-lowering treatments were used by 936 (8.4%) of participants and of those, 278 (29.7%) were still using them after 18 months, thus unlikely to have any effect on our results. Finally, we emphasize the distinction between lipid associations with dementia/CD and the absence of effect modification by statin treatment, consistent with our findings and previous studies.

In conclusion, we observed an association between LDL levels, but not statin treatment, in participants with T2D, and incident dementia/CD. Although causality cannot be determined by our study, the results are in line with multiple randomised controlled trials. However, to understand the long-term effects of lipid levels and statin treatment on dementia/CD, studies of longer follow-up are still needed.

## Supplementary Information

Below is the link to the electronic supplementary material.

Supplementary Material 1

## Data Availability

Restrictions apply to the availability of these data, which were used by agreement of the ADVANCE Management Committee for the current study and are not publicly available..

## Abbreviations

ADVANCE: The Action in Diabetes and Vascular Disease: Preterax and Diamicron Modified Release Controlled Evaluation
BP: Blood pressure
CD: Cognitive decline
eGFR: Estimated glomerular filtration rate
HDL: High-density lipoprotein
LDL: Low-density lipoprotein
MMSE: Mini Mental State Examination
T2D: Type 2 diabetes
