# Supplementary material for: Lipoprotein levels and statin treatment related to dementia and cognitive decline in individuals with type 2 diabetes: an observational analysis from the ADVANCE study
Source: Cardiovasc Diabetol. 2025 Aug 18;24:340. doi: 10.1186/s12933-025-02894-3 (PMC12362958; doi:10.1186/s12933-025-02894-3)
Supplement: Supplementary file 1 — Supplementary Material 1 [file 12933_2025_2894_MOESM1_ESM.docx]

Lipoprotein levels and statin treatment related to dementia and cognitive decline in individuals with type 2 diabetes: An observational analysis from the ADVANCE study

Supplementary table 1. Analysis of randomized treatment effect on cognitive decline or dementia for participants by statin treatment at baseline and for participants by statin treatment initiation during the first 18 months of the study period, respectively.

|  | Blood pressure treatment effect (active vs placebo) | | Glucose treatment effect (intensive vs standard) | |
| --- | --- | --- | --- | --- |
|  | **OR (95% CI)** | ***P* for interaction** | **OR (95% CI)** | ***P* for interaction** |
| Statin treatment at baseline |  |  |  |  |
| No | 1.00 (0.88-1.12) | 0.320 | 1.03 (0.92, 1.16) | 0.072 |
| Yes | 0.89 (0.73-1.08) |  | 0.84 (0.68, 1.02) |  |
| Statin treatment initiation during the first 18 months of the study |  |  |  |  |
| No | 0.99 (0.87-1.12) | 0.677 | 1.01 (0.89-1.15) | 0.392 |
| Yes | 1.06 (0.77-1.47) |  | 1.18 (0.85-1.63) |  |

Supplementary table 2. Absolute risk of (A) dementia/cognitive decline and (B) dementia during follow-up for participants with statin treatment vs no statin treatment (reference) at baseline.

|  | Adjusted for age and sex | Fully adjusted |
| --- | --- | --- |
| Dementia or cognitive decline |  |  |
| Without statin treatment at baseline | 17.0% | 17.0% |
| With statin treatment at baseline | 14.7% | 15.1% |
| Dementia |  |  |
| Without statin treatment at baseline | 0.75% | 0.51% |
| With statin treatment at baseline | 0.61% | 0.52% |

Absolute probabilities were estimated using multinomial logistic regression with the competing risk of death adjusted for age and sex, and additionally for region of residence, age at completion of highest level of education, randomised treatment group, baseline Mini Mental State Examination score, type 2 diabetes duration, waist circumference, smoking status, alcohol intake, systolic blood pressure, estimated glomerular filtration rate, urinary albumin-creatinine ratio, serum low-density lipoprotein cholesterol, and serum glycated haemoglobin. Predicted probabilities for each outcome category were generated for individuals with and without statin use, setting all covariates to their mean values.

Of 11,140 participants included in analysis, 1,827 (16.4%) had the outcome dementia or cognitive decline, and 109 (1.0%) had the outcome dementia.

Supplementary figure 1. Flow chart of measurements used in the current study.

Abbreviations: MMSE, mini-mental state examination.

Supplementary figure 2. Flow chart of participants in the study and data used for (A) primary and (B) secondary analysis.

Abbreviations: ADVANCE, The Action in Diabetes and Vascular Disease: Preterax and Diamicron Modified Release Controlled Evaluation.

Supplementary figure 3. Forest plot of odds ratio and 95% CI of (A) dementia/cognitive decline and (B) dementia during follow-up for participants with statin treatment vs no statin treatment (reference) at baseline overall and by subgroups after adjustments for age and sex.


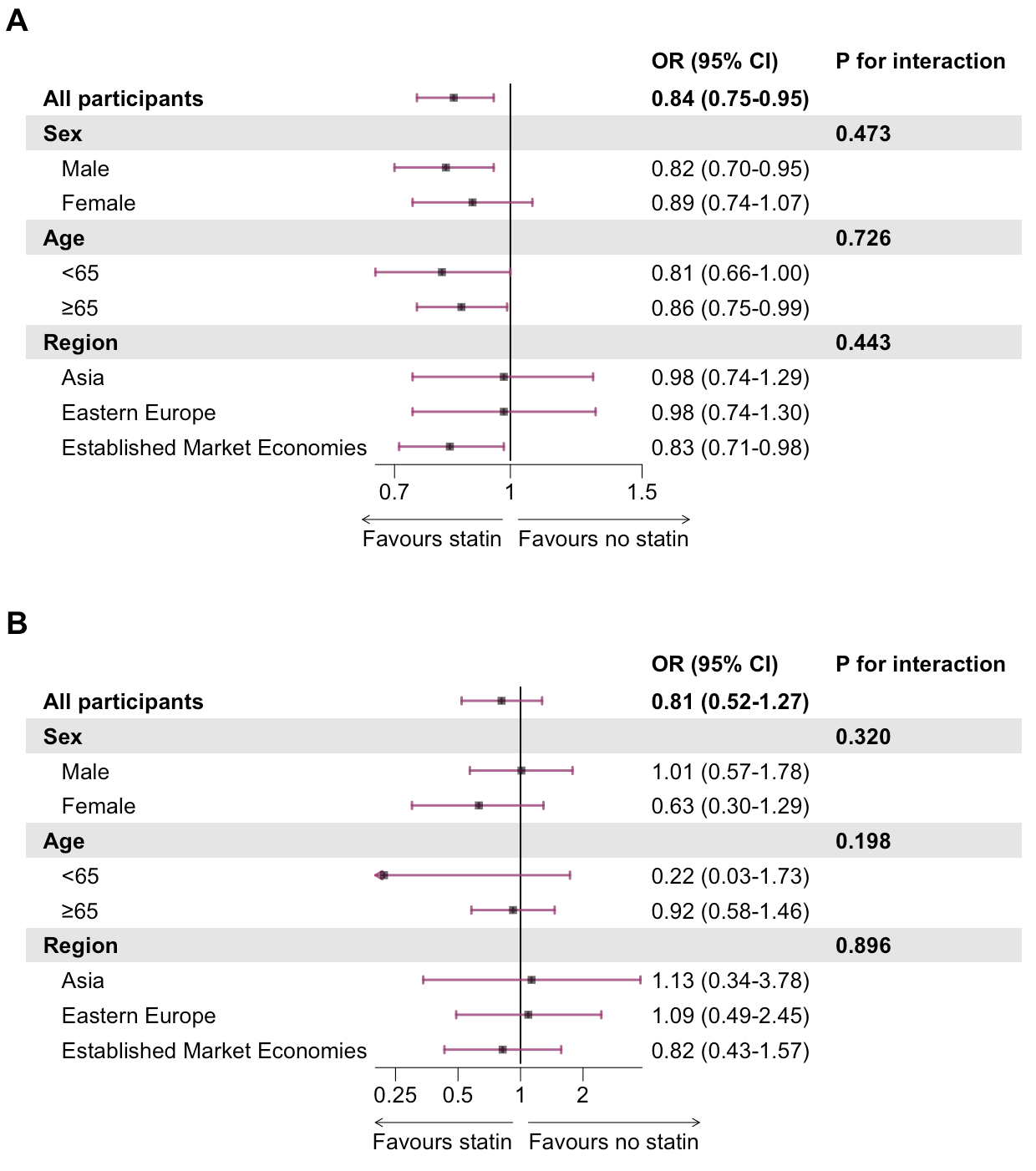


Multinomial logistic regression with the competing risk of death. For analysis of male and female participants, respectively, adjustment for sex was omitted.

Of 11,140 participants included in analysis, 1,827 (16.4%) had the outcome dementia or cognitive decline, and 109 (1.0%) had the outcome dementia.

Supplementary figure 4. Forest plot of odds ratio and 95% CI of (A) dementia/cognitive decline and (B) dementia during follow-up per standard deviation increase in standardized baseline total cholesterol, low-density lipoprotein cholesterol, and high-density lipoprotein cholesterol, respectively, in participants without statin treatment at baseline after adjustments for age and sex.


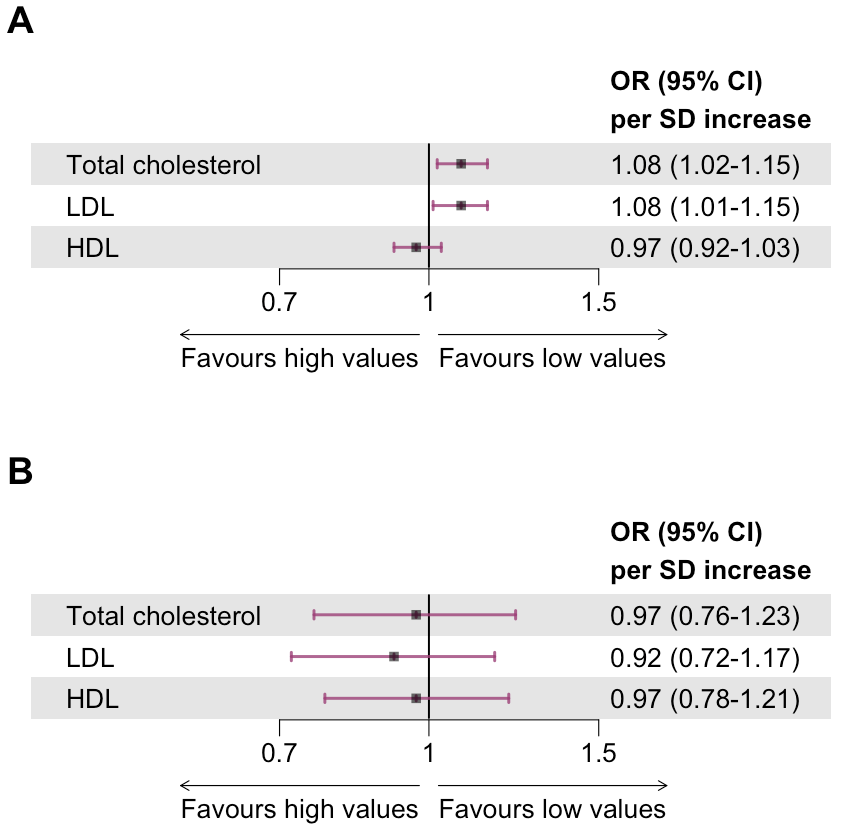


A multinomial logistic regression model was fitted to evaluate the association between baseline cholesterol levels and competing risks of death. Baseline cholesterol levels were standardized before analysis (mean-centred and divided by the standard deviation) to facilitate interpretation of results. Adjustments were for age and sex.

Of 7994 participants without statin treatment at baseline included in analysis, 1363 (17.1%) had the outcome dementia or cognitive decline, and 83 (1.0%) had the outcome dementia.

Abbreviations: HDL, high-density lipoprotein; LDL, low-density lipoprotein.

Supplementary figure 5. Forest plot of odds ratio and 95% CI of (A) dementia/cognitive decline and (B) dementia during follow-up per standard deviation increase in standardized baseline total cholesterol, low-density lipoprotein cholesterol, and high-density lipoprotein cholesterol, respectively, in all participants after multiple adjustment.


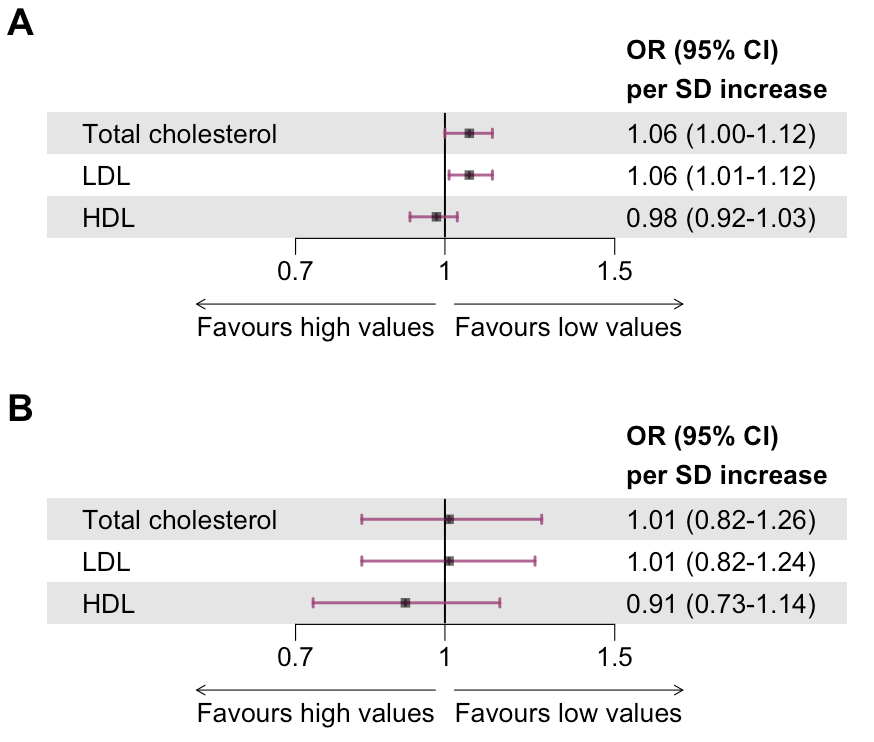


A multinomial logistic regression model was fitted to evaluate the association between baseline cholesterol levels and competing risks of death. Baseline cholesterol levels were standardized before analysis (mean-centred and divided by the standard deviation) to facilitate interpretation of results. Adjustments were for age, sex, region of residence, age at completion of highest level of education, randomised treatment group, baseline Mini Mental State Examination score, type 2 diabetes duration, waist circumference, smoking status, alcohol intake, systolic blood pressure, estimated glomerular filtration rate, urinary albumin-creatinine ratio, statin treatment at any time during the study period, and serum glycated haemoglobin. *P* for interaction between statin treatment at baseline and total cholesterol, LDL, and HDL, was 0.027, 0.110, and 0.022, respectively, for dementia/cognitive decline, and 0.302, 0.087, and 0.302, respectively, for dementia.

Of 11,140 participants without statin treatment at baseline included in analysis, 1827 (16.4%) had the outcome dementia or cognitive decline, and 109 (1.0%) had the outcome dementia.

Abbreviations: HDL, high-density lipoprotein; LDL, low-density lipoprotein.

Supplementary figure 6. Forest plot of odds ratio and 95% CI of (A) dementia/cognitive decline and (B) dementia during follow-up per standard deviation increase in standardized baseline total cholesterol, low-density lipoprotein cholesterol, and high-density lipoprotein cholesterol, respectively, in all participants after adjustments for age and sex.


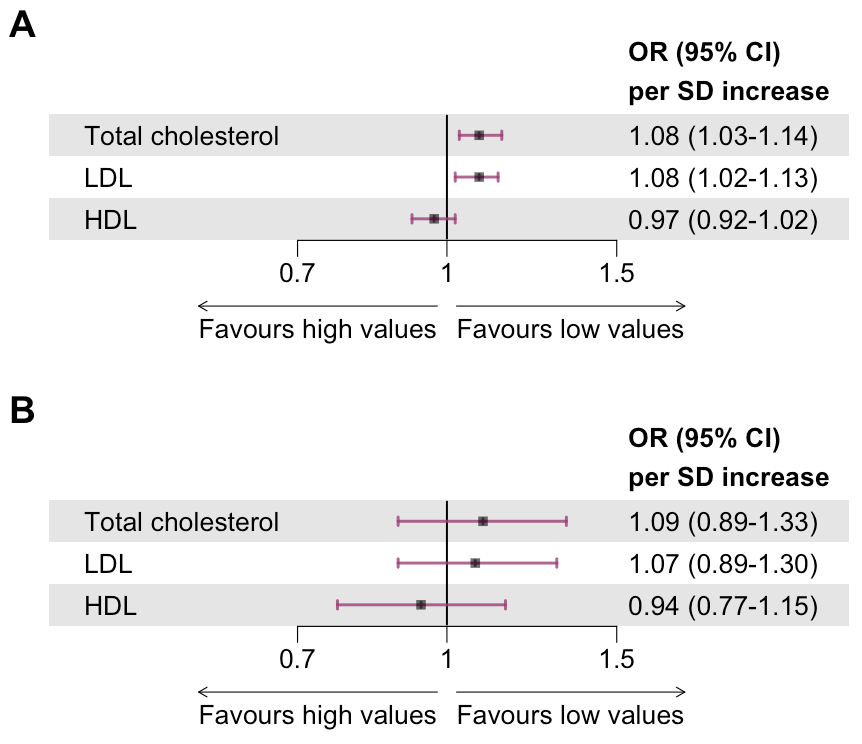


A multinomial logistic regression model was fitted to evaluate the association between baseline cholesterol levels and competing risks of death. Baseline cholesterol levels were standardized before analysis (mean-centred and divided by the standard deviation) to facilitate interpretation of results. Adjustments were for age and sex. *P* for interaction between statin treatment at baseline and total cholesterol, LDL, and HDL, was 0.465, 0.324, and 0.500, respectively, for dementia/cognitive decline, and 0.080, 0.010, and 0.531, respectively, for dementia.

Of 11,140 participants without statin treatment at baseline included in analysis, 1827 (16.4%) had the outcome dementia or cognitive decline, and 109 (1.0%) had the outcome dementia.

Abbreviations: HDL, high-density lipoprotein; LDL, low-density lipoprotein.

Supplementary figure 7. The absolute risk of each outcome category illustrated using predicted probabilities from multinomial logistic regression with total, LDL, and HDL cholesterol modelled as natural cubic splines with four degrees of freedom. Models were unadjusted (panels A through C), adjusted for age and sex (panels D through F), and fully adjusted (panels G through I).

Values outside the 0.1^st^ to 99.9^th^ percentiles were excluded. Full adjustments were for age, sex, region of residence, age at completion of highest level of education, randomised treatment group, baseline Mini Mental State Examination score, type 2 diabetes duration, waist circumference, smoking status, alcohol intake, systolic blood pressure, estimated glomerular filtration rate, urinary albumin-creatinine ratio, statin treatment at any time during the study period, and serum glycated haemoglobin.
